# Comparison of risedronate versus placebo in preventing anastrozole-induced bone loss in women at high risk of developing breast cancer with osteopenia

**DOI:** 10.1016/j.bone.2019.04.016

**Published:** 2019-07

**Authors:** Ivana Sestak, Glen M. Blake, Rajesh Patel, Robert E. Coleman, Jack Cuzick, Richard Eastell

**Affiliations:** a Centre for Cancer Prevention, Wolfson Institute of Preventive Medicine, Queen Mary University London, Charterhouse Square, London EC1M 6BQ, UK; b School of Biomedical Engineering & Imaging Sciences, King's College London, St Thomas' Hospital, London SE1 7EH, UK; c Imperial College London, London SW7 2AZ, UK; d Department of Oncology and Metabolism, University of Sheffield, Western Bank, Sheffield S10 2TN, UK

**Keywords:** Osteopenia, Bone mineral density, Anastrozole, Risedronate, Bone marker, Breast cancer risk

## Abstract

Anastrozole has been shown to prevent breast cancer in postmenopausal women at high risk of the disease, but has been associated with substantial accelerated loss of bone mineral density (BMD) and increased fractures. Here, we investigate the effect of risedronate on BMD after 5 years of follow-up in the IBIS-II prevention trial. 1410 women were enrolled in the bone sub-study and stratified into three strata according to the lowest baseline T-score at spine or femoral neck. The objective was to compare the effect of oral risedronate (35 mg weekly) versus placebo in osteopenic women in stratum II who were randomised to anastrozole in the main study. 258 osteopenic, postmenopausal women at high risk of developing breast cancer for whom baseline and follow-up bone mineral density measurements were available. 5-year mean BMD change at the lumbar spine for osteopenic women randomised to anastrozole and risedronate was −0.4% compared to −4.2% for those not on risedronate (*P* < 0.0001) but not significantly different between risedronate users and non-users at the hip (*P* = 0.2). 5-year mean PINP change was −20% for those randomised to anastrozole and risedronate compared to 3% for those not on risedronate but on anastrozole (*P* < 0.0001). Our results confirm the bone loss associated with the use of anastrozole and show that anastrozole-induced BMD loss in the spine can be controlled with risedronate treatment. However, our results suggest that weekly oral risedronate is unable to completely prevent anastrozole induced bone loss at the hip.

## Highlights

• Results confirm anastrozole induced bone mineral density decrease in women at increased risk of developing breast cancer
• Risedronate counterbalances the negative effect of anastrozole induced bone mineral density loss at the lumbar spine
• Long-term intake of risedronate did not prevent bone loss at the total hip in osteopenic women
• N-Terminal Propeptide of Type I Collagen (PINP) can predict bone mineral density changes

## 1 Introduction

Postmenopausal women are at high risk of developing osteoporosis (low mineral bone density (BMD)) due to decreasing levels of estrogen. Early postmenopausal BMD loss is estimated to be between 1 and 3% per year at the spine and 1 to 2% per year at the hip [1]. Bisphosphonates, which increase BMD by inhibiting osteoclast-mediated bone resorption [2,3], can prevent bone loss in postmenopausal women.

Aromatase inhibitors (AIs) have become the standard adjuvant treatment option for postmenopausal women with hormone receptor positive breast cancer. The risk of BMD loss, and therefore fractures, in this patient population is increased due to AIs ability to suppress estrogen levels by inhibiting the conversion of androgens to estrogens by the aromatase enzyme in soft tissues, especially fat. The majority of studies investigating the effect of AIs on BMD have been performed in postmenopausal women with early breast cancer receiving adjuvant tamoxifen as a comparison group, which has been shown to have a beneficial effect on BMD [4,5]. Most studies demonstrating a beneficial effect of bisphosphonates on the bone have been performed in breast cancer patients receiving an AI [[6], [7], [8], [9], [10]], and little is known about the effect of bisphosphonates in healthy postmenopausal women who are at risk of developing the disease.

The IBIS-II trial compared anastrozole with placebo in postmenopausal women at high risk of developing breast cancer and found a significant 53% reduction in breast cancer with anastrozole [11]. Due to these results, anastrazole has been recommended by the National Institute for Health and Care Excellence (NICE) for the prevention of breast cancer for postmenopausal women with family history. We have previously reported that 3 years of oral risedronate can prevent BMD loss in osteopenic and osteoporotic postmenopausal women who were receiving anastrozole [12]. Furthermore, this was the first study to report the effect of anastrozole on BMD loss in healthy postmenopausal women in a placebo-controlled trial. The decrease in BMD and the increase in bone turnover markers with aromatase inhibition would be expected to be associated with an increase in fracture risk, although our study wasn't powered to test for this. Here, we update the IBIS-II bone sub-study results by adding in the bone turnover marker N-Terminal Propeptide of Type I Collagen (PINP) and report in detail on the effect of risedronate on BMD in postmenopausal women with osteopenia.

## 2 Materials and methods

### 2.1 Study design and participants

**Fig. 1:**
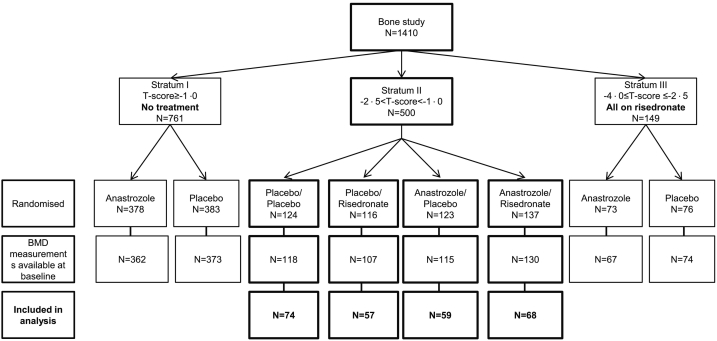
CONSORT diagram of enrolled women and BMD data included in this analysis.

We have previously described the study design and eligibility of the IBIS-II bone sub-study [12]. Entry criteria were designed to include women aged 45–60 years who had a relative risk of breast cancer that was at least two times higher than in the general population, those aged 60–70 years who had a risk that was at least 1.5 times higher, and those aged 40–44 years who had a risk that was four times higher. The IBIS-II bone sub-study enrolled 1410 women and they were stratified into three groups according to the lowest baseline T-score at either femoral neck or lumbar spine (Fig. 1).

Women who were osteopenic (−2.5 ≤ T-score < −1.0) were entered into stratum II and were additionally randomised to receive oral risedronate (35 mg per week) or matching placebo (*N* = 500). For this analysis only women from stratum II and those who did not develop breast cancer or died were included. All women were advised, but not required, to take vitamin D and calcium supplements. Exclusion criteria for the bone sub-study included previous bilateral hip fractures, women with any type of metabolic bone disease, and women who have regularly taken medication affecting bone metabolism within the past 12 months prior to study entry. Breast cancer development was the primary endpoint of the IBIS-II trial and women were excluded from the current analysis if they developed breast cancer at any point during the active treatment period. The trial was approved by the UK North West Multicentre Research Ethics Committee and was done in accordance with the Declaration of Helsinki, under the principles of good clinical practice. Participants provided written informed consent.

### 2.2 Assessments

BMD was assessed by follow-up DXA scans at the lumbar spine and hip at 1, 3 and 5 years. BMD measurements at the femoral neck are more variable as it relies on a smaller region of the bone than total hip. Therefore, we report here BMD measurements for the total hip. A final DXA scan at 7 years (two years after treatment cessation) was also performed, but these results are not reported here. T-scores were calculated using either the GE-Lunar [13] or Hologic [14] manufacturer's reference age-specific ranges for the lumbar spine (L1 to L4) and the NHANES III reference range for the femoral neck region [15]. For quality assurance, all baseline and follow-up DXA scans were reviewed centrally by two clinical scientists with expertise in bone densitometry (GMB and RP). Women in stratum II of the bone sub-study were randomised to receive oral risedronate or matching placebo using randomly chosen blocks of size six, eight, or ten to maintain balance. Compliance in stratum II was determined full if women took their allocated risedronate at least 80% over 5 years of follow-up (at least 208 weeks over 5 years). Women who had a BMD loss of 6% or more at the 12 month's visit were furthermore required to have a safety DXA scan at 24 months of follow-up. Similarly, those with a BMD loss of over 10% at 36 months had a safety scan at 48 months, and those with a BMD loss of over 16% at the 60 month's visit, had an interval scan at 72 months. Further details on assessments in the bone sub-study can be found in our previous publication [12].

Blood samples were taking in the non-fasting state and the serum separated and stored at −70C. The total N-Terminal Propeptide of Type I Collagen (PINP) was measured using the Cobas e411 automated immunoassay (Roche Diagnostics, Penzburg, Germany). The precision of PINP was assessed by measuring a serum quality control sample daily. The coefficient of variation was calculated and expressed as a percentage. The mean (SD) PINP concentration of this QC was 47.7 (2.4) ng/ml and the coefficient of variance was 5%.

### 2.3 Statistical analysis

The primary objective of this analysis was to compare the effect of risedronate versus placebo on the BMD change between baseline and 5 years at both the lumbar spine and total hip for women in stratum II who were randomised to anastrozole. Secondary objectives included comparison of baseline and randomised treatment effects on PINP measurements at study entry and 12 and 60 months of follow-up.

All results are expressed as percent mean BMD changes (with corresponding 95% confidence intervals) at the lumbar spine and total hip between baseline and 5 years. BMD changes and differences between treatment groups were assessed using paired *t*-tests for normal distribution. Pearson's correlation coefficient analysis was used to examine the relationship between and the values of BMD and PINP. Adverse events were compared with relative risks. *P*-values were two-sided, based on normal approximation and all confidence intervals were at the 95% level. Analyses were performed using STATA version 13.1 (College Station, Texas USA).

## 3 Results

1410 postmenopausal women were entered into the bone sub-study (Fig. 1). 258 women in stratum II had baseline and subsequent follow-up BMD measurements up to 5 years and were included in this analysis. Baseline characteristics for women in this analysis are shown in Table 1.

**Table 1:** Baseline characteristics for osteopenic women according to main and risedronate randomisation.

	P/P (N = 74)	P/R (N = 59)	A/P (N = 57)	A/R (N = 68)
Age (years), median (IQR)	59.7 (56.8–63.3)	60.8 (57.4–61.4)	60.2 (55.2–65.3)	59.7 (55.9–64.2)
BMI (kg/m^2^), median (IQR)	26.8 (23.6–32.0)	26.2 (24.5–29.4)	26.3 (23.9–30.7)	26.2 (23.3–26.7)
Previous HRT use (%)	43.2%	42.4%	45.6%	51.5%
Never smokers (%)	59.5%	64.4%	61.4%	58.8%
Lowest baseline T-score at lumbar spine or femoral neck, median (IQR)	−1.34 (−1.78 to −1.11)	−1.78 (−2.03 to −1.41)	−1.38 (−1.85 to −1.09)	−1.73 (−2.10 to −1.33)
PINP, median (IQR)	54.1 (40.7–69.1)	56.2 (40.7–71.1)	52.4 (42.6–70.4)	54.0 (45.6–67.5)

Data are median (interquartile range) or percentage (%). Abbreviations: P = Placebo, R = Risedroante, A = Anastrozole, IQR = interquartile range, BMI = body mass index, kg = kilogram, m = meter, HRT = hormone replacement therapy, PINP = N-Terminal Propeptide of Type I Collagen.

Overall, compliance to risedronate in stratum II was very good and only 26 women (4%) were identified as not fully compliant over 5 years of follow-up. Sensitivity analyses excluding these women did not alter the results presented here (data not shown). The main reason of missing DXA data was due to withdrawal from the main IBIS-II study due to anastrozole related adverse events. The most common reported adverse event were joint-related symptoms, such as arthralgia, joint stiffness, and joint pain. Most of these events were reported within the first 2 years since randomisation. Other reasons for non-compliance in the bone sub-study were the development of breast cancer (primary endpoint of the main trial), other cancers, and death. Women who developed breast cancer or those who died were not included in this analysis. All women were advised to take calcium and vitamin D supplements but no specific doses were specified or required as per protocol. 68% of women took these supplements during the 5-year treatment period. We did not observe any difference in BMD changes between those on supplements compared to those who did not take them, irrespective of treatment allocation.

**Fig. 2:**
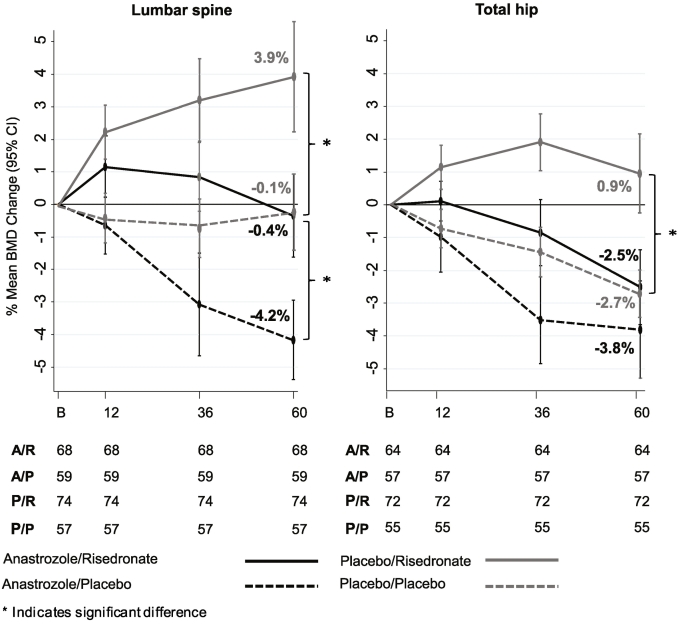
Mean % BMD changes at lumbar spine and total hip at each follow-up visit for women in stratum II. Numbers below the figures show women at each follow-up visit with available DXA scan.

127 osteopenic women who were randomised to anastrozole in the main trial had all BMD measurements available between baseline and 5 years of follow-up. Of these 68 were additionally randomised to risedronate (versus 59 to placebo). We observed a significant difference in mean % BMD change for those on risedronate (−0.4%) at the lumbar spine after 5 years of follow-up compared to those not receiving risedronate (−4.2%) (Difference: −3.8% (95% CI -5.5 to −2.3); *P* < 0.0001) (Fig. 2).

Osteopenic women receiving anastrozole and placebo showed a linear decrease in lumbar spine BMD with time over 5 years of follow-up whereas those receiving anastrozole and risedronate showed an initial BMD increase at the lumbar spine, which stabilised within 5 years of follow-up (Fig. 2). At the total hip, risedronate did not prevent anastrozole-induced bone loss (Fig. 2). Women receiving risedronate had a mean BMD decrease at the total hip of −2.5% compared to a mean decrease of −3.8% for those not receiving risedronate (Difference: −1.3% (95% CI -3.2 to 0.5); *P* = 0.2). The BMD decrease observed at the total hip with anastrozole and risedronate (−2.5%) was very similar to that among osteopenic women not receiving any treatment (−2.7%, Fig. 2).

Osteopenic women who were randomised to placebo in the main trial and additionally to risedronate (*N* = 74) showed a mean BMD increase of 3.9% (95% CI 2.2 to 5.6) at the lumbar spine over 5 years of follow-up (Fig. 2). A highly significant difference (*P* = 0.0001) was observed compared to those receiving placebo (*N* = 57) who maintained their mean BMD at the lumbar spine over 5 years of follow-up (−0.1% (95% CI -1.3 to 1.0)). A similar but less striking picture was seen at the total hip. Women receiving risedronate (*N* = 72) showed an initial increase in mean BMD after 3 years (1.8% (95% CI 1.0 to 2.7), which declined to 0.9% (95% CI -0.3 to 2.1)) after 5 years. However, these BMD changes were significantly different in those receiving placebo only (*N* = 55) (−2.7% (95% CI -3.4 to −2.0); *P* < 0.0001) (Fig. 2). Overall, most rapid bone loss occurred in year 1, as defined by BMD loss of >6% since baseline, occurred in year 1 (*N* = 17). These women had to stop with the trial and were given a bisphosphonate. 1 women had rapid bone loss at year 5, which was defined as BMD change of >16% since baseline.

**Fig. 3:**
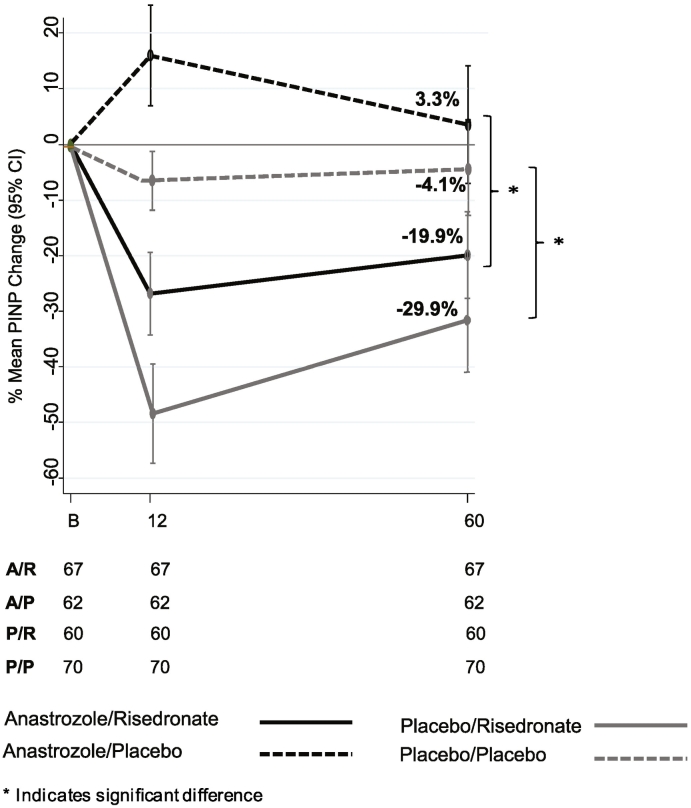
Mean % PINP changes between baseline and 12, 60 months for women in stratum II receiving anastrozole (left) or placebo (right) and randomised to either risedronate or placebo. Numbers below the figures show women at each follow-up visit with available DXA scan.

Women receiving anastrozole and risedronate had a mean PINP decrease of 27% at 12 months and 20% at 60 months as compared to a mean increase of 16% at 12 months and 3% at 60 months for those not receiving risedronate (Fig. 3).

Women receiving risedronate only had a mean PINP decrease of 48% at 12 months and 30% at 60 months as compared to a mean decrease of 6% at 12 months and 4% at 60 months for those not receiving risedronate (Fig. 3). All differences were highly statistically significant (*P* < 0.001). The 12-month change in PINP in the risedronate group was correlated with 12-month change in lumbar spine BMD (*r* = −0.33, *P* = 0.0003) and in the total hip (*r* = −0.21, *P* = 0.03). The 60-month change in PINP in the risedronate group was correlated with 60-month change in lumbar spine BMD (*r* = −0.26, *P* = 0.008) and in the total hip (*r* = −0.29, *P* = 0.003). There were no significant correlations between change in PINP and BMD in the groups not receiving risedronate.

Pre-defined side effects, such as abdominal pain, bloating, diarrhoea, and constipation, reported during 5 years of follow-up were statistically not significantly different between risedronate treatment allocations (data not shown). We also did not observe any osteonecrosis of the jaw during 5 years of risedronate treatment. A total of 97 osteopenic women reported fractures in the bone sub-study (Table 2) but no significant difference between anastrozole and placebo was observed (RR = 0.98 (95% CI 0.61–1.56); *P* = 0.7). No difference in reported fractures was observed between women receiving risedronate compared to those who did not (Table 2).

**Table 2:** Number of women reporting fractures according to treatment allocation.

	Anastrozole	Placebo	OR (95% CI)
Fractures	50	47	0.98 (0.61–1.56)
No risedronate	30	29	1.02 (0.57–1.83)
Risedronate	20	18	0.91 (0.46–1.81)

Data are numbers or Odds Ratio (OR). Abbreviations: OR = Odds Ratio, CI = Confidence Interval.

## 4 Discussion

In postmenopausal women with osteopenia, this study showed that weekly oral risedronate for 5 years can prevent anastrozole-induced bone loss at the lumbar spine, but not the hip. Risedronate was very well tolerated and no serious adverse events, such as osteonecrosis of the jaw, were reported during the 5 years on treatment. Compliance to risedronate was very good and the exclusion of non-compliant women had no impact on BMD changes.

Apart from the MAP.3 trial [16], most evidence that AIs have a negative effect on BMD come from adjuvant trials in women with early breast cancer [4,17,18]. These trials have all shown significant BMD loss with an AI, but have in common that the comparator was tamoxifen, which has been shown to have a beneficial impact on bone [9,19]. Similarly, all other reports that have investigated the effect of a bisphosphonate on BMD in women taking anastrozole come from treatment trials [8,[20], [21], [22], [23]]. Greenspan et al. found that weekly risedronate improved BMD and decreased bone turnover markers in postmenopausal women with hormone receptor positive breast cancer who were given an AI for 2 years. Although this was a small study and the follow-up period was relatively short, they demonstrated that measurements of bone markers (C-telopeptide crosslinks type I collagen (CTX) and N-Terminal Propeptide of Type I Collagen (PINP)) can predict BMD changes. We observed correlations between 12-month change in PINP and BMD that were very similar.

One key finding of our study was the observation that long-term risedronate intake wasn't preventing bone loss at the total hip, although it was effective at the lumbar spine. We did not find any evidence that poor compliance with risedronate was responsible for this. Risedronate might not be sufficiently potent in the presence of long-term anastrozole used, which is supported by a lesser effect at 60 months on the PINP changes. Furthermore, our current results differ from previous analysis at the three year follow-up [12]. Risedronate clearly is sufficiently potent to prevent hip bone loss in women not receiving anastrozole after 60 months, a finding in keeping with a clinical trial over 60 months in 220 women with osteoporosis [24]. We did not observe any reduction in fractures with risedronate irrespective of main randomisation. This is in contrast to adjuvant breast cancer studies, which have shown to significantly reduce the incidence of fractures with bisphosphonates, both vertebral and non-vertebral fractures [4,25,26]. However, these trials have all in common that the comparator was tamoxifen, which has been shown to have beneficial impact on bone [9,19,26]. Our trial was not designed to investigate the use of risedronate for the prevention of fractures and they were not our primary endpoint. In postmenopausal osteoporosis, the effect of risedronate on non-vertebral fracture reduction is about 20% [27]. In the current study, the point estimate of reduction in fracture risk was 9% but in order to detect such a change in fracture risk, a much larger study would need to be performed.

The results from this study in women at risk of developing breast cancer can be compared to previous studies of women with breast cancer treated with aromatase inhibitors. For example, in the ATAC study [28] the 5-year rate of bone loss at the lumbar spine and total hip were 6.1% and 7.2%, considerably greater than in this preventive study of 4.2% and 3.8%, respectively. It has been considered that women with breast cancer are particularly susceptible to the development of osteoporosis [29]. This is somewhat surprising given that the PINP level was similar in the ATAC study and IBIS-II (mean PINP around 55 μg/L) and the change in PINP was between 155 and 20% [19].

This analysis has several strengths and limitations. Strengths include a good sample size (*N* = 258) of women with osteopenia and long-term follow-up of 5 years. The population included in this analysis came from a large prevention trial [11] with excellent clinical records and detailed follow-up. An additional strength of this study is the evaluation of BMD changes and risedronate in a placebo-controlled trial of healthy postmenopausal women who are at increased risk of developing breast cancer. Our results might be useful for women at increased risk of breast cancer who consider taking anastrozole for prevention purposes.

Limitations of our study include the incomplete set of BMD data at 5 years, which was mainly due to withdrawal from the main IBIS-II study due to anastrozole related side effects. Therefore, our results might not be representative for the whole study population. However, when all BMD measurements (incomplete data between baseline, 1, 3, and 5 years) were included in a sensitivity analysis, similar results were observed. Two types of DXA machines were used to assess BMD changes in this study. However, comparisons of BMD changes between Hologic and GE-Lunar scanners did not reveal any significant differences. DXA measurement doesn't take into account bone structure and micro-architecture of the bone, which plays an important role in determining bone strength [16,30,31]. However, it was not possible to assess these structural changes within the remit of the bone sub-study protocol. All women had to stop with bone anabolic therapy or selective estrogen replacement therapy 12 months before joining the IBIS-II trial. Although we did not collect detailed information on medication before the 12 months to trial entry, we do not believe that prior intake of these drugs had any impact on BMD changes during the trial. Lastly, we were not able to measure a bone resorption marker as the only one that can be measured in serum after long-term storage and is reliable is CTX, which needs to be measured in a sample obtained fasting. The reason we measured PINP is that estrogen deficiency results in an increase both in resorption and formation markers (due to coupling) and bisphosphonates result in a decrease in both types of marker.

## 5 Conclusion

The IBIS-II results strongly support anastrozole for preventive treatment of high risk postmenopausal women [11]. This updated analysis in healthy postmenopausal women in a placebo controlled trial confirmed the beneficial effect of risedronate on bone loss at the lumbar spine only in osteopenic women receiving anastrozole. We found that the use of a bisphosphonate and monitoring of bone density by DXA scans can control BMD loss induced at the lumbar spine by anastrozole in the preventive setting. However, bone loss at the hip was not completely prevented and therefore other more potent bisphosphonates (e.g. alendronate, zoledronic acid) for prevention of hip bone loss and fracture need to be investigated in this setting. Risedronate was well tolerated and may provide a therapeutic option to maintain skeletal health in women at increased risk of developing breast cancer.
